# Cross-validation and modifications of the perceived mattering questionnaire—Physical education in Chinese inservice physical education teachers

**DOI:** 10.3389/fpsyg.2022.982565

**Published:** 2022-08-16

**Authors:** Xin Zhang, Jingwen Liu, Xiaolu Liu, Rulan Shangguan, Xiaofen D. Hamilton, Jianmin Guan, Dwan Bridges

**Affiliations:** ^1^Department of Physical Education, Nanjing University of Aeronautics and Astronautics, Nanjing, China; ^2^Department of Kinesiology, California State University, Fullerton, CA, United States; ^3^Department of Kinesiology and Health, Georgia State University, Atlanta, GA, United States; ^4^School of Physical Education, South China University of Technology, Guangzhou, China; ^5^Department of Curriculum & Instruction, University of Texas at Austin, Austin, TX, United States; ^6^Department of Kinesiology, University of Texas at San Antonio, San Antonio, TX, United States; ^7^Department of Kinesiology, Nutrition & Food Science, California State University, Los Angeles, CA, United States

**Keywords:** revalidation, perceived mattering questionnaire, physical education teachers, validity, reliability

## Abstract

This study aimed to provide evidence of validity and reliability for the Perceived Mattering Questionnaire-Physical Education (PMQ-PE) developed by Richards et al. (2017) for the Chinese physical education (PE) teachers. The PMQ-PE consisted of two factors (i.e., PE matters and PE teacher matters) with four items in each, measuring four dimensions (attention, importance, dependence, and ego-extension). PMQ-PE in Chinese (PMQ-PEC) was validated among 1,278 elementary and secondary school PE teachers in China, of whom 59.0% were secondary PE teachers and 70.2% were males. Confirmatory factor analysis (CFA) with the entire sample found a poor model fit. Then exploratory factor analysis (EFA) using half of the sample indicated there was only one factor in PMQ-PEC. CFA of the other half of the sample suggested a one-factor model with the elimination of three unfitted items showed a better fitness to the data. Cronbach's alpha value was also acceptable. The final version of PMQ-PEC included five items with one factor that demonstrated acceptable validity and reliability and was deemed acceptable among Chinese PE teachers after the above modifications were made.

## Introduction

It has been reported that less than one-third of states required high school physical education (PE) and there was only one state that required PE for all K-12 programs in the US (Dauenhauer et al., 2019). This means that PE received less attention in US schools. Although similar data in other countries were unavailable, the marginalization of PE exists in many countries (Johns and Dimmock, 1999; Gray et al., 2012; Burnett, 2021). This has particularly resulted from the implementation of standardized testing, which focused on core subject matters such as math, science, and language (Gray et al., 2012; Richards et al., 2014). Because PE is not included in these tests with limited resources (Burnett, 2021), it is considered as a peripheral or less important subject matter in schools (Lux and McCullick, 2011; Richards et al., 2019). More alarmingly, Sheehy (2011) stated:

The marginalization of PE is a complex issue that continues to overshadow the unique contribution that quality PE can make to the lives of young people. The marginal status of PE has become the status quo, while PE teachers, for the most part, have failed to engage in practices that would combat this situation. (p. 42)

Because of the marginalization of PE in educational settings, the perceived mattering PE teachers hold regarding their jobs and roles in the school has become an important strand of research in recent years (Gaudreault et al., 2018; Richards et al., 2019). Previous research on the topic has denoted that PE teachers experienced the effects of marginalization in their daily teaching (Washburn et al., 2020), resulting in negative feelings associated with marginality causing stress, burnout, and early career retention (Whipp et al., 2007; O'Halloran and Moynihan, 2020). On the other hand, due to the well-known potential contribution of PE to help students adopt a healthy lifestyle, many countries such as Austria (Dollman et al., 2006), China (Chinese Department of Sports Hygiene Health Education, 2014), Finland (Mäkelä et al., 2014), Scotland (Gray et al., 2012), South Africa (Burnett, 2021), and the US (McKenzie, 2019) have paid more attention to quality PE (QPE) in conjunction with health education in schools. As such, there is an urgent need to generate new knowledge about perceived mattering and its effects on QPE. Unfortunately, research on the perceived mattering of PE has been rare and only a handful of studies have been available in the literature and most were conducted in the US (e.g., Marshall, 2001; Gaudreault et al., 2018; Richards et al., 2019; O'Halloran and Moynihan, 2020).

### Definition of perceived mattering

Moschella and Banyard (2021) noted that “mattering is the subjective perception that others are aware of us, care about us, and depend on us at the interpersonal (e.g., friends) and societal levels (e.g., workplace, school)” (p. 55). Richards et al. (2017) pointed out that perceived mattering is the construct that others are aware of oneself, or the tendency to believe one is important to other people. In the current study, mattering was defined as “the perception that, to some degree and in any of a variety of ways, we are a significant part of the world around us” (Elliott et al., 2004, p. 339). The reason for choosing this definition is that it captures the essence of perceived mattering – the people in the specific environment (schools in our study) that affect one's work.

### Perceived mattering and the perceived mattering questionnaire—PE (PMQ-PE)

#### Dimensions

A thorough examination of current literature on mattering indicated that researchers agreed upon four dimensions: attention, importance, dependence, and ego-extension, focusing on the people in the teaching context (Richards et al., 2017; Gaudreault et al., 2018). It is important to point out that all four dimensions are not physical but psychological tendencies (Marshall, 2001). A number of scholars (Richards et al., 2017, 2018a; Gaudreault et al., 2018; Washburn et al., 2020) agreed that attention means that a person or subject is of interest to other people or groups. Importance is related to the concept that others value the contributions of a person or subject to the group. Dependence refers to the degree that others or the group rely on a person or subject, while ego-extension is associated with the notion that others care about the successes or failures of a person or subject.

#### PMQ-PE

Because PE teachers' perceived mattering can only be thoroughly investigated with reliable and valid instruments, it is important to develop a scale for measuring PMQ-PE that can be used with confidence. The PMQ-PE developed by Richards et al. (2017) has shown evidence of reliability and validity in examining PE teachers' perceived mattering in the US. The PMQ-PE includes two factors (PE matters and PE teacher matters) with four items for each, underpinning the concept that individuals' perceptions of PE as a subject matter in schools are different from that of PE teachers. Specifically, “PE matters” refers to PE teachers' perceived mattering concerning PE as the subject matter in schools. PE teacher matter focuses on PE teachers' perceptions on their matter to people in schools (Richards et al., 2017; Washburn et al., 2020). The items were set to a 4-point Likert scale with 1 representing “not at all,” 2 representing a little, 3 representing “somewhat,” and 4 representing “a lot.” “PE matters” refers to PE teachers' perceived mattering concerning PE as the subject matter in schools. PE teacher matter focuses on PE teachers' perceptions that they matter to people in schools (Richards et al., 2017; Washburn et al., 2020). No negatively worded items were used. The PMQ-PE was validated using 460 PE teachers and has demonstrated acceptable reliability (Cronbach's alphas for the PE teacher matters and PE matters were 0.87 and 0.86, respectively) and construct validity (NFI = 0.97, CFI = 0.98, SRMR = 0.03, RMSEA = 0.07) (Note: the cut-off value for RMSEA to be acceptable was smaller than 0.05 according to Meyers et al., 2017). Convergent, discriminant, and divergent validity were also tested using confirmatory factor analysis (CFA) and all the values were within the acceptable range (see Richards et al., 2017 for more detailed information). To date, a few studies have used PMQ-PE to quantitatively examine PE teachers' perceived mattering in the US (Richards et al., 2018a,b, 2020; O'Halloran and Moynihan, 2020; Washburn et al., 2020).

Because the curricula, instructional time, and academic status of PE vary greatly across countries, it is important to validate PMQ-PE in the country of an intended research site before the scale can be used with confidence. To the best of our knowledge, however, this scale has not been validated in the Chinese population. Given that the marginalization of PE occurs worldwide, there is a need to validate PMQ-PE in Chinese PE teachers (PMQ-PEC) in order to help explore perceived mattering issues in China and regions where Chinese is the official language. The cross-culture validated scale would help facilitate research on perceived mattering in PE across nations. This may eventually lead to further understanding of the entire issue concerning the marginalization of PE in different countries to provide baseline data for changing the status of PE in schools so that students can be better physically educated. Therefore, the purpose of the study was to test the reliability and validity of PMQ-PEC in China, where marginalization of PE occurs, even though PE is given more scores in standardized tests and gaining more governmental attention. It is hoped that the present study would stimulate more revalidation studies on the topic in other countries where marginalization of PE occurs to help PE teachers improve the quality of their professional careers.

## Methods

In alignment with the process outlined by Richards et al. (2017) study on validating the PMQ-PE in the US, this investigation duplicated the validation process. An Institutional Review Board (IRB) approval was not obtained because it does not have such a requirement in China (Keating et al., 2020). However, the guidelines for human subject studies used in the US were closely followed to protect the privacy of participants. No identifiable personal information was collected in the current study.

### Participants

A convenient sample of 1,278 full-time PE teachers in elementary and secondary schools in China participated in the study. Although the survey is anonymous, the online survey tool can still generate information concerning where the participants were physically located. The participants were from all Chinese provinces and autonomous cities except Taiwan. There were 70.2% of males and 29.8% of females, which is about the same as the population of PE teachers in China in which about two-thirds of the population are males (Keating et al., 2020). In addition, 511 (40.0%) and 715 (55.9%) were elementary and secondary PE teachers, respectively (Note: missing 4.1%). The average age was 35.3 (*SD* = 8.3).

### PMQ-PE in Chinese (PMQ-PEC)

In an effort to cross-validate the PMQ-PE and explore potential convergent and discriminant validity of the PMQ-PE with Chinese teachers, the PMQ-PE was translated into Chinese first by the first author and then edited by the entire research team. The PMQ-PEC was sent to five Chinese-born professors in the Department of Kinesiology at American public state universities to ensure the accuracy of the translation. Minor changes were made to the wording in the Chinese version of the instruments based on the feedback. Specifically, PMQ-PEC also consisted of two factors (PE matters and PE teacher matters), consisting of the original four items in each domain. Instead of using a 4-point scale in PMQ-PE, however, PMQ-PEC used a 7-point Likert response format for an adequate survey sensitivity to measure the perceptions of participants (Finstad, 2010). The responses ranged from 1 (strongly disagree) to 7 (strongly agree). The point of 4 measured the neutral perception. Demographic information such as age, gender, the total years of teaching, and the educational level of teaching was surveyed at the end of the PMQ-PEC.

### Data collection

A Chinese online survey tool, namely Wenjuanxing, a professional and most widely used survey website in China, was used to deliver the survey so that it could be completed using either a computer or a cellphone. The snowballing method was used to generate a relatively large sample size by sending the survey link to PE teachers and encouraging them to forward the link to other PE teachers. All participants were asked to provide their affiliations and universities that issued their degrees to ensure the authenticity of the data collected. Information about the name of the schools and years of teaching at each educational level was also checked to make sure that the participants were indeed PE teachers.

### Data analysis

Based on the purpose of the study, CFA using the entire sample was first run to test the model proposed by Richards et al. (2017) with the two factors (i.e., PE matters and PE teacher matters). Because the high correlations between factors and low factor loadings of items were found, it was deemed necessary to re-examine the structure of PMQ-PEC in the sample of Chinese PE teachers. As such, the sample was randomly divided into two sub-samples (i.e., sample A and sample B) with a relatively close sample size (see Table 1 for the demographic information of the samples). Exploratory factor analysis (EFA) was used to examine the structure of the data using sample A, followed by CFA using sample B. Data screening, descriptive statistics, and the EFA were conducted using IBM SPSS 23.0 software while IBM AMOS 26 was used for running CFA (IBM Corporation, 2021).

**Table 1:** Demographic information of the samples.

**Variables**	**Entire sample**	**Sample A for EFA**	**Sample B for CFA**			
	**M (*SD*)**	***N* (%)**	**M (*SD*)**	***N* (%)**	**M (*SD*)**	***N* (%)**
Age	35.3 (8.3)		35.1 (8.2)		35.6	
Year of teaching						
Elem.	3.8 (7.0)		3.7 (6.9)		3.8 (7.0)	
Secon.	7.5 (9.3)		7.2 (9.0)		7.8 (9.7)	
Gender						
Male		898 (70.2)		460 (72.0)		438 (68.5)
Female		381 (29.8)		179 (28.0)		201 (31.5)
School level						
Elementary		511 (40.0)		256 (40.1)		255 (39.9)
Secondary		715 (55.9)		361 (56.5)		354 (55.4)
Missing		53 (4.1)		22 (3.4)		30 (4.7)

#### EFA

EFA was run using maximum likelihood extraction to test the underlying factor structure of the PMQ-PEC. Specifically, factors with eigenvalues over one were extracted, and a direct oblimin rotation was selected due to the high correlation between the two factors. Items with factor loading values >0.30 were kept (Meyers et al., 2017). Significant cross-loading items were viewed as unfitted and should be removed.

#### CFA

CFA was then performed to confirm the factor structure. The convergent, divergent, and discriminant validity of the PMQ-PEC was also examined using the second sub-sample. Cronbach's alpha was computed to assess the internal consistency of scores produced by PMQ-PEC. The cut-off value for alpha was set at alpha >0.70 to show acceptable reliability (Meyers et al., 2017). The homogeneity of each domain was first tested by fitting the data to a one-factor model. Then a two-factor model was used to examine its two-factor structure (i.e., PE matters and PE teacher matters). According to Meyers et al. (2017), the following five absolute and incremental fit measures were employed to evaluate the fitness of the data: (a) standardized root mean square residual (SRMR), (b) comparative fit index (CFI), (c) Tucker-Lewis Index (TLI), which is also called the non-normed fit index, (d) goodness of fit index (GFI), and (e) root mean square error of approximation (RMSEA). In general, the chi-square index is a statistically based measure of goodness-of-fit, and is very sensitive to sample size and violation of normality (Meyers et al., 2017; Richards et al., 2017). Given the sample size in the current study is considered large, it is very likely that the chi-square index will be significant (Richards et al., 2017; Fan et al., 2018). As such, the chi-square index was not used as one of the model fit indices. For model fit, CFI, TLI, and GFI values need to be >0.95 while RMSEA and SRMR need to be <0.05 to be deemed acceptable. Moreover, structure coefficients should be >0.30 to be included in the factor (Meyers et al., 2017). More importantly, professional knowledge should also be used to assess the model fit when some of the fit indices are not within the acceptable range (Keating and Silverman, 2004).

##### Convergent and discriminant validity

CFA was also used to examine convergent and discriminant validity. Following the methods used in the study of Richards et al. (2017), the absolute value of factor loadings, composite reliability values (ρ_c_), and average variance extracted (AVE) scores were selected to assess convergent validity. The convergent validity is acceptable when (a) factor loadings are above 0.50 and significant (Brown, 2006), (b) ρ_c_ values are ≥0.70 (Diamantopoulos and Siguaw, 2000), and (c) AVE scores are larger than 0.50 (Fornell and Larcker, 1981). Discriminant validity was tested using $\sqrt{AVE}$. Discriminant validity is acceptable if $\sqrt{AVE}$ for each of the latent variables is higher than the correlations between the latent variables (Teo et al., 2009).

## Results

Because CFA using the entire sample, EFA using sample A, CFA and correlational analysis using sample B were sequentially completed, the results section was reported in the order in which it was finished. The results of EFA were taken into consideration when CFA was conducted. The correlational analysis was influenced by the CFA results (i.e., unfitted items were eliminated).

### Construct validity

#### CFA with the entire sample

**Figure 1:**
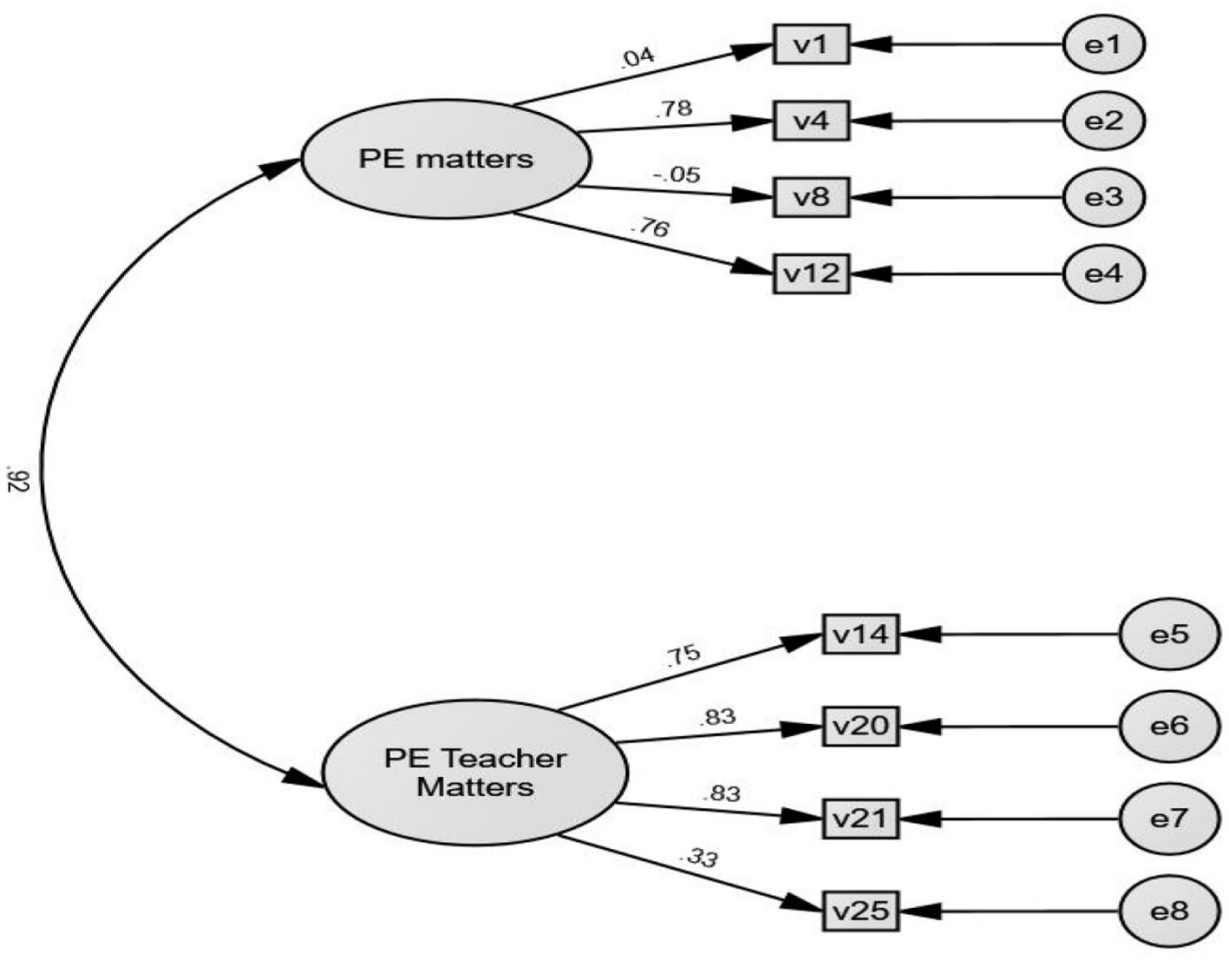
Two factors with eight items for CFA.

Although the fit indices based on the two-factor model were within the acceptable range (CFI = 0.97, GFI = 0.95, TLI = 0.95, RMSEA = 0.07, SRMR = 0.02), the correlation between the two factors was 0.92 (see Figure 1), indicating multicollinearity and poor discriminant validity. Furthermore, the standardized regression weights of items V1, V8, and V25 were <0.50, suggesting insignificant contribution to their corresponding factor (Meyers et al., 2017; Richards et al., 2017). As such, it was deemed necessary to re-examine the structure of PMQ-PEC.

#### EFA using one of the sub-samples

The EFA results indicated there was only one item in the PE teacher matters (i.e., V21) with a factor loading smaller than 0.7 (see Table 2). The total variance explained was 66.5%. There was only one eigenvalue >1. When forced to have two factors using EFA, no clear structure was identified.

**Table 2:** Factors and items in PMQ-PEC and factor loadings of EFA using sample A.

**Factors**	**Item No**.	**Items**	**Factor loading**
PE matters	V1	How interested are other people, generally, in physical education at your school?	0.83
	V12	How much attention do you feel other people pay to physical education at your school?	0.84
	V4	How important do you feel physical education is to other people at school?	0.85
	V8	How much do you feel other people at school would miss physical education if it went away?	0.86
PE teacher matters	V20	How important do you feel you are to other people at school?	0.86
	V25	How interested are other people, generally, in what you have to say at school?	0.86
	V14	How much attention do you feel other people pay to you at school?	0.89
	V21	How much do you feel other people at school would miss you if you went away?	0.43

#### CFA using the other sub-sample with two different models

CFA was run using one factor with eight items. The indices for model fit were within the acceptable range (CFI = 0.99, GFI = 0.96, TLI = 0.96, RMSEA = 0.06, SRMR = 0.03). However, the coefficients (i.e., standardized regression weights) for three items (i.e., V1, V8, and V25) were still <0.50. As such, the three items were deleted.

**Figure 2:**
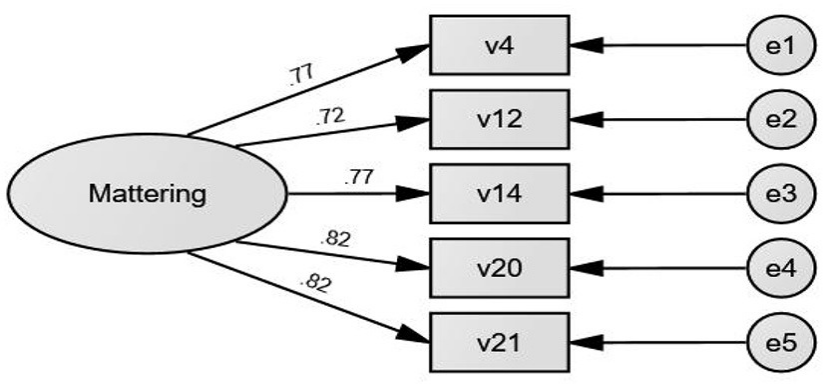
CFA model of one factor with 5 items.

The model fit with the remaining five items revolved on one factor was examined using CFA again. All coefficients were >0.5, indicating that the items were all reasonably robust as good indicators of the underlying factors (Richards et al., 2017). Most of the fit indices were acceptable (CFI = 0.97, GFI = 0.97, TLI = 0.95, SRMR = 0.02) except RMSEA, which was >0.05 (RMSEA = 0.11) (see Figure 2). Cronbach's alpha was calculated with the five items and it was acceptable (alpha = 0.88). Taken together, the data were a good fit to the model (Meyers et al., 2017).

### Convergent, discriminant validity, and correlational analysis

As noted earlier, convergent validity is assessed in the following three ways: (a) factor loadings, (b) ρ_c_ values, and (c) AVE scores. The final model had all factor loadings >0.5 (see Table 3). ρ_c_ value was 0.90, which was greater than the cut-off value (0.70) and AVE score was 0.64 while the acceptable value is 0.50. Discriminant validity was explored using $\sqrt{AVE}$. The value of $\sqrt{AVE}$ was 0.80 while the correlation between PMQ-PEC, role stressors, and resilience was 0.36 (*p* < 0.001), and 0.14 (*p* < 0.001), respectively.

**Table 3:** The modified PMQ-PEC and factor loadings of the one-factor CFA using sample B.

**Factors**	**Item No**.	**Items**	**Factor loading**
Mattering	V4	How interested are other people, generally, in physical education at your school?	0.83
	V12	How much attention do you feel other people pay to physical education at your school?	0.86
	V14	How important do you feel physical education is to other people at school?	0.92
	V20	How much do you feel other people at school would miss physical education if it went away?	0.88
	V21	How much do you feel other people at school would miss you if you went away?	0.50

## Discussion and conclusions

It has been well-documented that PE as a subject matter in schools has been marginalized worldwide (Whipp et al., 2007; Richards et al., 2018b; O'Halloran and Moynihan, 2020; Spicer and Robinson, 2021) and PE teachers did not receive the respect they deserved (Whipp et al., 2007), even though researchers suggested that PE programs have the potential to help students adopt a healthy lifestyle (McKenzie and Sallis, 1991; Dollman et al., 2006; Sallis et al., 2012). This situation hinders our endeavor to combat childhood obesity and illnesses caused by sedentary lifestyles. As suggested in the literature, more efforts are needed to improve the quality of PE and increase the status of PE in schools (McKenzie and Lounsbery, 2014).

Although many factors may have contributed to the marginalization of PE, the lack of valid and reliable instruments measuring PE teachers' perceived mattering certainly did not help us combat marginalization. More alarmingly, while marginalization occurs globally, no studies have examined PE teachers' perceptions of mattering from an international perceptive. The current study filled in the above gap in the literature and provided a means for quantitatively investigating this matter in different countries to shed new light on reducing marginalization. More studies on the reliability and validity of the PMQ-PE in different countries are needed in the future so that our confidence in measuring the perceived mattering of PE teachers across nations would be increased.

### The ratio of sample size to the item number

Although the rule of thumb for the ratio of sample size to the number of items in a questionnaire is usually 5:1, and no samples are completely reflective of the population (Meyers et al., 2017), the larger the ratio the better the study is in general (Keating et al., 2008; Fan et al., 2018). The ratio of the sample size to the item number was about 158:1. As such, the sample size used in the current study was deemed sufficient.

### Modifications that may improve the validity and reliability of PMQ-PEC

According to Richards et al. (2017), PMQ-PE was valid and reliable using a sample of in-service PE teachers in the US. However, PMQ-PEC did not show acceptable construct validity given that the two factors were strongly correlated (*r* = 0.92) and the factor loadings for three items were smaller than 0.5. Therefore, modifications were made to improve the model fit. As a result, there was only one factor with five items in the final version of PMQ-PEC. This result may be associated with the cultural difference between the US and China. The subject matter of PE in schools may be strongly connected to PE teachers in China while the two are viewed separately in the US. Unfortunately, to the best of our knowledge, no empirical data are available to confirm the above contention. More studies on the topic are needed in the future.

The elimination of three unfitted items may also suggest that only the two dimensions (i.e., attention and importance) would affect the perceived mattering among Chinese PE teachers. The other two dimensions (i.e., dependence and ego-extension) may not apply to the Chinese population. The reason may be that Chinese PE teachers are seldom asked to take important roles in their school affairs, leaving few opportunities for them to have many interactions with other personnel in schools. As such, they are less likely to perceive dependence and ego-extension. Of more importance, many PE teachers are coaches while after-school sport programs play an important role in the US (Eyler et al., 2019; Hodges et al., 2021). However, sports do not have the same role as that in the Chinese educational system, contributing less to the development of schools in China. Therefore, the dimensions of dependence and ego-extension may not be applicable to the Chinese PE teachers. Overall, the modifications resulted in shortening the length of the questionnaire, providing more advantages to recruit more participants considering that shorter surveys tend to generate a larger sample size (Groves et al., 2011). Not only does the modified version of PMQ-PEC fit in the Chinese sample, but it also saves more time in completing the survey.

### Convergent and discriminant validity

While the assessment method for convergent validity was the same as that used by Richards and colleagues, the discriminant validity was tested differently (Richards et al., 2017) because only one factor was included in PMQ-PEC. As a result, it is inappropriate to compare the results found in the current study to those reported by Richards et al. (2017). Future studies should focus on the examination of the discriminant validity of PMQ-PEC.

Therefore, the final version of PMQ-PEC was deemed that there was a good fit of the data by CFA because the GFI, TLI, SRMR, and CFI were within the acceptable range. The RMSEA was only slightly above the acceptable value. In addition, the factor loading for each item was >0.50, indicating that all remaining items were robust indicators of the factor they were targeted to measure (Meyers et al., 2017). The Cronbach's alpha was >0.8, which was within the acceptable range.

## Limitation

It is important to point out that all data collection was completed online. It is unknown if such a data collection method could have any effects on the validation of PMQ-PEC. Future research should compare the reliability and validity of survey using the data collected in person with the paper-pencil version of the survey.

## Conclusions and implications for future research

Instead of having two factors and eight items, the modified PMQ-PEC consists of one factor with five items that measure one's perceived mattering of PE and PE teachers. Construct validity, convergent validity, discriminant validity, and reliability were established based on CFA results, AVE and ρ_c_ values, and correlational analysis results.

The availability of a valid and reliable measure of PMQ-PEC will allow researchers to investigate important issues concerning the perceived mattering of PE and PE teachers. Furthermore, by calculating the average score of the questionnaire, international comparison studies on the topic may be conducted to shed new light on the shared and unique causes of marginalization of PE between schools in US and China. More research using representative samples is needed in the future to further examine the reliability and validity of the scores generated by PMQ-PEC.

## Data availability statement

The raw data supporting the conclusions of this article will be made available by the authors, without undue reservation.

## Ethics statement

Ethical review and approval was not required for the study on human participants in accordance with the local legislation and institutional requirements. Written informed consent for participation was not required for this study in accordance with the national legislation and the institutional requirements.

## Author contributions

XZ took the main responsibility for the research design, data collection, and structured the method part of the manuscript. JL and XL were mainly responsible for data analyses, result interpretation, and looking for updated literature. XH participated in the literature search and provided her expertise in the data analyses. JG and DB mainly contributed to the manuscript preparation, revision in terms of results, and discussion. RS was responsible for coordinating the project, participated in data collection, and writing the discussion. All authors contributed to the article and approved the final version.

## Conflict of interest

The authors declare that the research was conducted in the absence of any commercial or financial relationships that could be construed as a potential conflict of interest.

## Publisher's note

All claims expressed in this article are solely those of the authors and do not necessarily represent those of their affiliated organizations, or those of the publisher, the editors and the reviewers. Any product that may be evaluated in this article, or claim that may be made by its manufacturer, is not guaranteed or endorsed by the publisher.

## References

[B1] Brown T. (2006). Confirmatory Factor Analysis for Applied Research. No location only publisher for 7^th^ edition. Guilford Press.

[B2] Burnett C. (2021). A national study on the state and status of physical education in South African public schools. Phys. Educ. Sport Pedagogy 26, 179–196. 10.1080/17408989.2020.1792869

[B3] Chinese Department of Sports Hygiene Health Education (2014). The Chinese Youth Fitness Testing Standards. Available online at: http://www.moe.gov.cn/s78/A17/twys_left/moe_943/moe_947/201407/t20140721_172364.html

[B4] Dauenhauer B. Keating X. Stoepker P. Knipe R. (2019). State physical education policy changes from 2001 to 2016. J. School Health 89, 485–493. 10.1111/josh.12757 30919961

[B5] Diamantopoulos A. Siguaw J. A. (2000). Introducing LISREL: A Guide for the Uninitiated. SAGE.

[B6] Dollman J. Boshoff K. Dodd G. (2006). The relationship between curriculum time for physical education and literacy and numeracy standards in South Australian primary schools. Eur. Phys. Educ. Rev. 12, 151–163. 10.1177/1356336X06065171

[B7] Elliott G. Kao S. Grant A.-M. (2004). Mattering: Empirical validation of a social-psychological concept. Self Identity 3, 339–354. 10.1080/13576500444000119

[B8] Eyler A. A. Piekarz-Porter E. Serrano N. H. (2019). Pay to play? State laws related to high school sports participation fees. J. Public Health Manage. Prac. 25, E27–E35. 10.1097/PHH.0000000000000813 29889175 PMC6416066

[B9] Fan Y. Keating X. D. Liu J. Zhou K. Shangguan R. Knipe R. (2018). Development of a scale measuring Chinese preservice physical education teachers' beliefs about the physical education profession. Asia-Pacific Educ. Res. 27, 365–372. 10.1007/s40299-018-0395-0

[B10] Finstad K. (2010). Response interpolation and scale sensitivity: evidence against 5-point scales. J. Usability Stud. 5, 104–110.

[B11] Fornell C. Larcker D. F. (1981). Evaluating structural equation models with unobservable variables and measurement error. J. Market. Res. 18, 39–50. 10.1177/002224378101800104

[B12] Gaudreault K. L. Richards K. A. R. Mays Woods A. (2018). Understanding the perceived mattering of physical education teachers. Sport Educ. Soc. 23, 578–590. 10.1080/13573322.2016.1271317

[B13] Gray S. MacLean J. Mulholland R. (2012). Physical education within the Scottish context: a matter of policy. Eur. Phys. Educ. Rev. 18, 258–272. 10.1177/1356336X12440019

[B14] Groves R. M. Fowler F. J. Jr. Couper C. Lepkowski J. M. Singer E. Tourangeau R. (2011). Survey Methodology. John Wiley & Sons.

[B15] Hodges M. Keating X. D. Smith S. (2021). Athletic directors' perceptions on pay-to-play. Appl. Res. Coach. Athletics Annual 36, 23–47.

[B16] IBM Corporation. (2021). IBM SPSS Statistics for Windows, Version 26.0. Armonk, NY: IBM Corporation.

[B17] Johns D. P. Dimmock IV C. (1999). The marginalization of physical education: impoverished curriculum policy and practice in Hong Kong. J. Educ. Policy. 14, 363–384. 10.1080/026809399286242

[B18] Keating X. D. Guan J. Ferguson Robert H. Chen L. Bridges D. M. (2008). Physical education teacher attitudes toward fitness tests scale: cross-revalidation and modification. Meas. Phys. Educ. Exerc. Sci. 12, 72–87. 10.1080/10913670801903969

[B19] Keating X. D. Liu J. Liu X. Colburn J. Guan J. Zhou K. (2020). An analysis of Chinese preservice physical education teachers' beliefs about the physical education profession. J. Teach. Phys. Educ. 1, 1–8. 10.1123/jtpe.2019-0095

[B20] Keating X. D. Silverman S. (2004). Physical education teacher attitudes toward fitness test scale: development and validation. J. Teach. Phys. Educ. 23:143. 10.1123/jtpe.23.2.143

[B21] Lux K. McCullick B. A. (2011). How one exceptional teacher navigated her working environment as the teacher of a marginal subject. J. Teach. Phys. Educ. 30, 358–374. 10.1123/jtpe.30.4.358

[B22] Mäkelä K. Hirvensalo M. Laakso L. Whipp P. R. (2014). Physical education teachers in motion: an account of attrition and area transfer. Phys. Educ. Sport Pedagogy 19, 418–435. 10.1080/17408989.2013.780590

[B23] Marshall S. K. (2001). Do I matter? Construct validation of adolescents' perceived mattering to parents and friends. J. Adolescence 24, 473–490. 10.1006/jado.2001.0384 11549327

[B24] McKenzie T. L. (2019). Physical activity within school contexts: the bigger bang theory. Kinesiol. Rev. 8, 48–53. 10.1123/kr.2018-0057

[B25] McKenzie T. L. Lounsbery M. A. F. (2014). The pill not taken: revisiting physical education teacher effectiveness in a public health context. Res. Q. Exerc. Sport 85, 287–292. 10.1080/02701367.2014.931203 25141081

[B26] McKenzie T. L. M. Sallis J. F. (1991). Physical education's role in public health. Res. Quart. Exercise Sport 62, 124–137. 10.1080/02701367.1991.10608701 1925034

[B27] Meyers L. S. Gamst G. Guarino A. J. (2017). Applied Multivariate Research: Design and Interpretation, 3rd Edn. SAGE. Available online at: https://us.sagepub.com/en-us/nam/applied-multivariate-research/book246895

[B28] Moschella E. A. Banyard V. L. (2021). Short measures of interpersonal and university mattering: evaluation of psychometric properties. J. Coll. Stud. Dev. 62, 55–71. 10.1353/csd.2021.0004

[B29] O'Halloran C. Moynihan C. (2020). Do personal accomplishment, resilience, and perceived mattering inhibit physical educators' perceptions of marginalization and isolation? JOPERD 91, 51–52. 10.1080/07303084.2020.1683391

[B30] Richards K. A. Gaudreault K. L. Woods A. M. (2017). Understanding physical educators' perceptions of mattering: Validation of the Perceived Mattering Questionnaire – Physical Education. Eur. Phys. Educ. Rev. 23, 73–90. 10.1177/1356336X16637320

[B31] Richards K. A. R. Gaudreault K. L. Starck J. R. Mays Woods A. (2018a). Physical education teachers' perceptions of perceived mattering and marginalization. Phys. Educ. Sport Pedagogy 23, 445–459. 10.1080/17408989.2018.1455820

[B32] Richards K. A. R. Gaudreault K. L. Woods A. M. (2018b). Personal accomplishment, resilience, and perceived mattering as inhibitors of physical educators' perceptions of marginalization and isolation. J. Teach. Phys. Educ. 37, 78–90. 10.1123/jtpe.2016-0228

[B33] Richards K. A. R. Templin T. J. Graber K. (2014). The socialization of teachers in physical education: review and recommendations for future works. Kinesiol. Rev. 3, 113–134. 10.1123/kr.2013-0006

[B34] Richards K. A. R. Washburn N. S. Hemphill M. A. (2019). Exploring the influence of perceived mattering, role stress, and emotional exhaustion on physical education teacher/coach job satisfaction. Eur. Phys. Educ. Rev. 25, 389–408. 10.1177/1356336X17741402

[B35] Richards K. A. R. Wilson W. J. Holland S. K. Haegele J. A. (2020). The relationships among perceived organization support, resilience, perceived mattering, emotional exhaustion, and job satisfaction in adapted physical educators. Adapted Phys. Activity Quart. 37, 90–111. 10.1123/apaq.2019-0053 31869818

[B36] Sallis J. F. McKenzie T. L. Beets M. W. Beighle A. Erwin H. Lee S. (2012). Physical education's role in public health: steps forward and backward over 20 years and HOPE for the future. Res. Q. Exerc. Sport 83, 125–135. 10.1080/02701367.2012.10599842 22808697 PMC6036633

[B37] Sheehy D. A. (2011). Addressing parents' perceptions in the marginalization of physical education. J. Phys. Educ. Recreation Dance 82, 42–56. 10.1080/07303084.2011.10598657

[B38] Spicer C. Robinson D. B. (2021). Alone in the gym: a review of literature related to physical education teachers and isolation. Kinesiol. Rev. 10, 66–77. 10.1123/kr.2020-0024

[B39] Teo T. Lee C. B. Chai C. S. Wong S. L. (2009). Assessing the intention to use technology among pre-service teachers in Singapore and Malaysia: a multigroup invariance analysis of the Technology Acceptance Model (TAM). Comput. Educ. 53, 1000–1009. 10.1016/j.compedu.2009.05.017

[B40] Washburn N. S. Richards K. A. R. Sinelnikov O. A. (2020). Investigating the relationships between perceived mattering, role stress, and psychological need satisfaction in physical education teachers. J. Teach. Phys. Educ. 39, 48–58. 10.1123/jtpe.2018-0342

[B41] Whipp P. R. Tan G. Yeo P. T. (2007). Experienced physical education teachers reaching their “use-by date:” Powerless and disrespected. Res. Quart. Exerc. Sport 78, 487–499. 10.5641/193250307X13082512817589 18274220

